# Quality of Life Outcomes in a Community Cohort of Adults With an Intellectual Disability Using the Personal Outcome Scale

**DOI:** 10.3389/fresc.2022.848492

**Published:** 2022-03-25

**Authors:** Tom Burke, Andrew Deffew, Owen Stafford, Caroline Docherty, Sandra Burke, Remco Mostert, Jos van Loon, Marco Lombardi, Marianne Vaughan, Robert Brickell, Mary Keogh, Wendy Mahon, David O'Halloran

**Affiliations:** ^1^KARE Services, Kilcullen, Ireland; ^2^School of Psychology, John Henry Newman Building, University College Dublin, Dublin, Ireland; ^3^School of Psychology, National University of Ireland Galway, Galway, Ireland; ^4^School of Psychology, University of Limerick, Limerick, Ireland; ^5^Hogeschool Ghent, Ghent, Belgium

**Keywords:** quality of life, intellectual disabilities, community-based services, personal outcome measures, psychological wellbeing

## Abstract

**Objectives:**

Quality of life (QoL) is a multi-dimensional phenomenon composed of core domains that are influenced by personal characteristics, values, and environmental contributions. There are eight core domains of QoL aligned with both the United Nations and the International Association for the Scientific Study of Intellectual and Developmental Disabilities (IASSIDD). The Personal Outcome Scale (POS), is a semi-structured self and proxy instrument that specifically measures these aspects of QoL for people with an intellectual disability.

**Methods:**

A total of 85 people with an intellectual disability and their primary keyworker (*n* = 85) took part in this study. A convenience sample recruitment strategy was employed to recruit participants during the calendar year from January–December 2020. Participants completed the self-report and proxy POS, and clinic-demographic data was also considered.

**Results:**

QoL is higher in those who have a dedicated service planner and also for those with a less severe to profound disability. People who were in gainful employment reported significantly higher QoL as did those availing of outreach and residential services, over and above local services.

**Conclusions:**

This research shows that there are distinct and specific factors that relate to QoL for people with an intellectual disability community-based services in Ireland. Future research could aim to investigate these longitudinally, and specifically how QoL relates to cognitive and functional outcomes.

## Introduction

Ensuring people with intellectual disabilities receive proper care and support in society requires a thorough consideration of the individual quality of life (QoL) since an intellectual disability has the potential to hinder one's independence, well-being, and ability to fully engage in the community (1). Fulfilling one's professional responsibilities in the field of intellectual disabilities involves understanding and applying best practices based on relevant conceptual models and frameworks regarding human functioning and disability, QoL, and individualized supports (2). Clinical practice and research in the field of intellectual disability have shown the importance of focusing on a person's QoL, and the mediating role that individualized supports can play in ameliorating the impact of one's disability, enhancing human functioning, and improving QoL overall (3). QoL is a multi-dimensional phenomenon composed of core domains that are influenced by personal characteristics, values, and environmental variables (4). Inherently, QoL is multifaceted and unique to an individual with some constructs that may resonate with many individuals and some with varying value and importance at the individual level (5, 6). As such, QoL can be a challenging concept to measure accurately, psychometrically (7).

There are eight core domains of QoL which are aligned with both the United Nations Convention on the Rights of Persons with Disabilities (4, 8) and with the Quality of Life Consensus Statement from the International Association for the Scientific Study of Intellectual and Developmental Disabilities (IASSIDD). The personal outcome scale [POS (9)], has been developed specifically to measure these eight aspects of QoL for people with an intellectual disability, as outlined in more detail below. These core domains of QoL are: personal development; self-determination; interpersonal relations; social inclusion; rights; mental well-being; physical well-being; and financial well-being (10). These eight core QoL domains, measured by the POS, have been assessed across several countries and cultures due to the potential impact of culture on QoL (11–13).

The POS assesses QoL using a semi-structured self-report interview format, as well as a secondary observer report which is used in conjunction with, and not in replacement of, the self-report. The POS is somewhat unique in its measurement of QoL for people with an intellectual disability, as it: (1) is based on a QoL-specific theoretical framework; (2) assesses personal outcomes with guided support in a semi-structured format; and (3) considers multi-informant reporting. To date, studies to test the reliability and validity of the POS have been conducted in the Netherlands (14), Portugal (15–18), Spain (19–21), and Italy (22). In terms of clinical outcomes within these studies, in Portugal it was found that living circumstances were related most to outcomes on the POS (16). Similarly, in the Netherlands, greater independence was associated with higher scores on the POS. As such, the first aim of this study was to investigate the psychometrics of the POS in an Irish sample, and secondly, to consider outcomes for people with an intellectual disability who attend a community-based service in Ireland. A final aim of this study was to investigate clinical- and service-based factors which may relate to QoL, as measured by the POS.

## Materials and Methods

### Setting and Sample

This study took place in a community-based service for people with an intellectual disability in Ireland. A total of 85 people who attend a community-based service for people with an intellectual disability and their primary keyworker (*n* = 85) were the participants. A convenience sample recruitment strategy was employed to recruit participants during the calendar year from January–December 2020. Within the host organization, members of the Quality Assurance Team (QAT: SB, MV, RB, MK, and WM) approached potential participants and informed them of the POS study, following ethical approval. Participants were then invited to take part and provided consent for their data to be used. Members of the QAT are not directly involved in the clinical care of the participants or service users, which may have reduced response bias, i.e., socially desirable responding. The mean age for the total group was 40.08 years ±14.20; 18.8% attended outreach services, 37.6% attended residential services, and 43.5% attended local (day) services. In brief, outreach services can be best described as a flexible and tailored support service for people who have an intellectual disability and high levels of independence, but lower support needs. Local services provide recreation, leisure, and specialized healthcare for people with an intellectual disability during the day. Local services support people with an intellectual disability to live in their communities and promote independent living. Residential services are provided for people with an intellectual disability who are unable to live at home, and they typically live there full-time.

Of the total cohort, 61% had a planner to support them; 52% of the group were in gainful employment at the time of completing the POS. There was a relatively equal gender distribution (52% female); 86% of the total cohort presented with a mild or moderate intellectual disability (29.5 and 56.5%, respectively). This research has been approved by the principal investigators host academic institution's research ethics committee (RECREF: HS-E-21-62).

### The Personal Outcome Scale

The personal outcome scale (9) was developed through an iterative process of expert consultation and focus groups with key stakeholders (including clients, family, direct support staff, and experts). The POS can be summed into a “Total Score” comprised of three subdomains (independence, social participation, and well-being), for both self-report and the observer/proxy report. Within these subdomains, there are 8 individual factors associated with the aforementioned QoL framework e.g., physical well-being, with an acceptable psychometric factor structure (19). These eight core domains of this model and measure have been assessed across several countries and cultures due to the potential impact of culture on QoL (11–13). Each QoL factor is broken down into corresponding domains. The Independence factor is broken down into personal development and self-determination domains; the Social Participation factor is broken down into interpersonal relations, social inclusion, and rights domains; and the Well-being factor is broken down into emotional, physical, and material well-being domains. There are six questions related to the domain presented under each section, resulting in a total of 48 questions. Under each question, the person is given 3 answers to choose from. They choose the most appropriate option depending on the extent to which each question applies to them. Under the self-determination domain of the self-report scale, for example, the question, “Can you decide not to do something asked of you?” is followed by the answer choices “always,” “sometimes,” or “seldom or never,” and the person may choose an answer based on their own experiences. The person's answers are then converted into scores using a 3-point Likert scale, with the total score out of a potential 144. The POS is always administered in both formats (self-report and observer/proxy report) to gather QoL data from both the subjective and objective perspectives.

### Data Processing and Analysis

Within the group, demographic characteristics were comparatively analyzed using independent samples *t*-tests with χ^2^ used for dichotomized variables, where relevant. MANOVA were used to compare multiple dependent variables (total self-report, total observer-report, and the cumulative total score). Classification for “good” internal validity, using Cronbach's alpha (a), remains at the internationally accepted value ≥0.71 and the acceptable was set at ≥0.6. Split-half reliability was also assessed using Spearman-Brown coefficient for equal length measures, to complement analyses of internal consistency. Correlations were used to investigate the relationship between self- and proxy-reported outcomes on the POS, and the relationship between age and outcomes. The threshold for statistical significance was set at *p* < 0.05. Statistical analyses were conducted using SPSS (Version 26.0).

## Results

### Scale Reliability and Correlates

**Figure 1:**
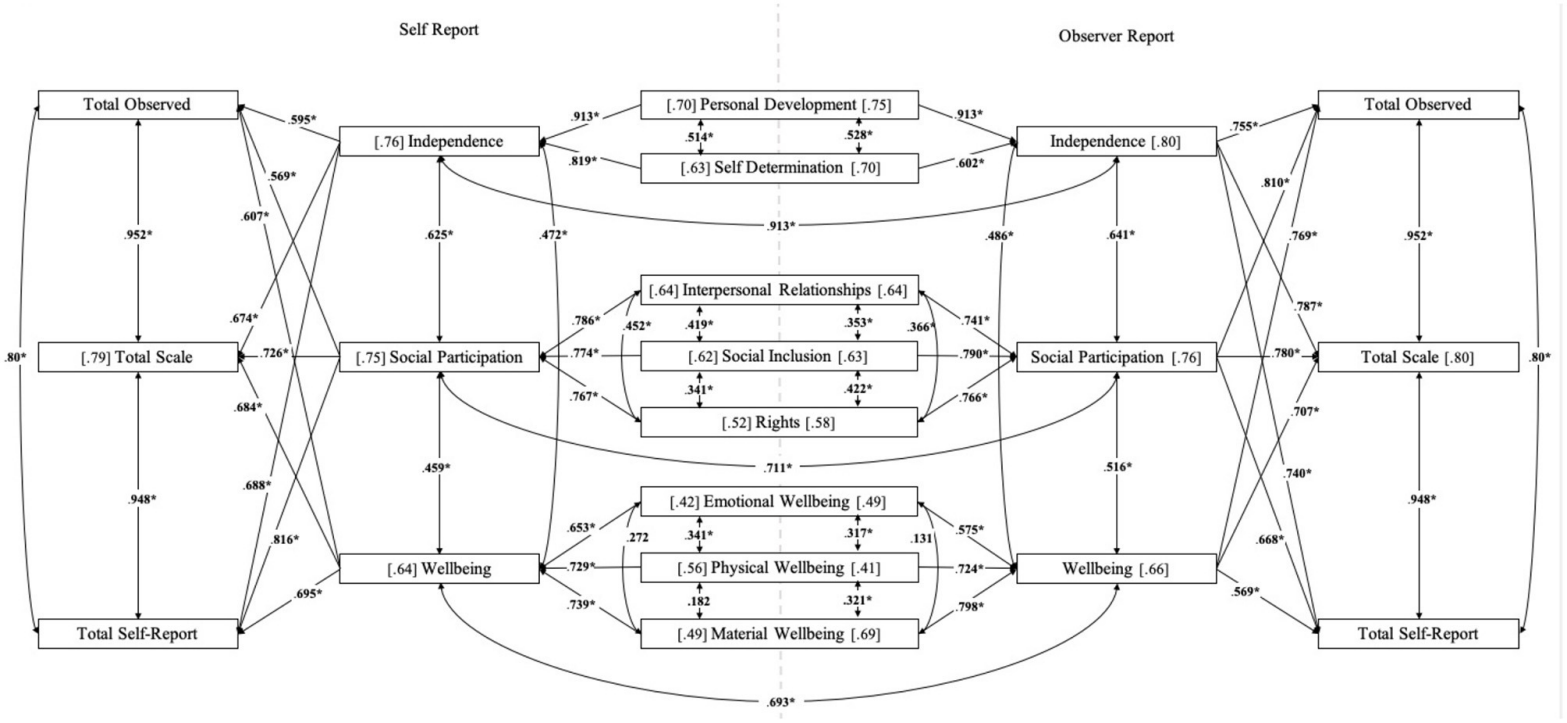
Outcomes relating to the [Cronbach's alpha] and between-within scale (correlations). The left side of the figure relates to the self-report POS outcomes, and the right, demarked by the dashed vertical line, relates to the observer report. Note, a Cronbach's alpha are reported in chain brackets [ ], and ≥60 is considered acceptable. Correlations are reported along the line which relates to the variables in question and significance is represented by *p ≤ 0.05.

Internal consistency was assessed using split-half reliability on the POS, with an unequal length analysis completed due to the varied number of items per subscale. Overall, the split-half reliability for the POS is 0.857. Scale reliability was completed by measuring the associated Cronbach's alpha on many levels. To investigate the reliability of the POS, the total observed, total self-reported, and a total scale (the summed total of the two aforementioned scales) were investigated. Furthermore, scales and subscales (a: independence, b: social participation, and c: wellbeing); and their subtotals (a: personal development and self-determination; b: interpersonal relationships, social inclusion, and rights; c: emotional wellbeing, physical wellbeing, and material wellbeing) were investigated. Figure 1 outlines the Cronbach's alpha and correlations of each of the subtests for both self and observer reports.

Considering the self-report first, there was a good scale validity reported for the total scale (*a* = 0.79), the independence (*a* = 0.76), and social participation (*a* = 0.75) subscales. The wellbeing scale (*a* = 0.64) was considered to have acceptable validity as a subscale. Each of the three subtotals within the wellbeing scale i.e., emotional wellbeing (*a* = 0.42), physical wellbeing (*a* = 0.56), and material wellbeing (*a* = 0.49), had reliability scales that would not be considered acceptable as unitary constructs. Similarly, the three constructs of interpersonal relationships (*a* = 0.64), social inclusion (*a* = 0.62), and rights (*a* = 0.52), which make up the social participation subscale, were below the recommended value of *a* = 0.7 for good reliability, with only the first two measures reaching an acceptable validity. Lastly, the independence subscale comprised of the personal development (*a* = 0.70) and self-determination (*a* = 0.63) subtotals, with both meeting the statistical threshold for acceptable reliability.

In terms of the between–within scale correlations, there were significant moderate to strong positive correlations between the total scale and the three subscales of independence (*r* = 0.674, *p* < 0.05), social participation (*r* = 0.726, *p* < 0.05), and wellbeing (*r* = 0.684, *p* < 0.05). There were also significant moderate positive correlations between the subscales, and between the subscales and subtotals, as seen in Figure 1. Of note, there were two non-significant correlations on the self-report outcomes. The first was between physical wellbeing and material wellbeing (*r* = 0.182); the second was between emotional wellbeing and material wellbeing (*r* = 0.272), despite each being contained within the wellbeing subscale. The remaining variable set was significantly correlated i.e., physical wellbeing and emotional wellbeing (*r* = 0.341, *p* < 0.05).

Regarding the observer-report and the scale validity, a similar pattern to the self-report was identified. The total scale score (*a* = 0.80) has good internal consistency, as did the independence (*a* = 0.80), and social participation (*a* = 0.76) subscales. Similar to the self-report, the wellbeing subscale (*a* = 0.66) had an acceptable, but not a good, validity. A similar pattern for the subtotals was also reflected with the wellbeing subscale i.e., emotional wellbeing (*a* = 0.49) and physical wellbeing (*a* = 0.41), achieving an unacceptable reliability coefficient. The material wellbeing subscale was reported to have acceptable psychometric properties (*a* = 0.69). The three constructs of interpersonal relationships (*a* = 0.64), social inclusion (*a* = 0.63), and rights (*a* = 0.58), which make up the social participation subscale, reached an acceptable, but not a good, validity. Lastly, the independence subscale comprised of personal development (*a* = 0.75) and self-determination (*a* = 0.70) subtotals, with both meeting the statistical threshold for acceptable reliability.

In relation to the between-within scale correlations for the observer-report, there were significant moderate to strong positive correlations between the total scale and the three subscales of independence (*r* = 0.787, *p* < 0.05), social participation (*r* = 0.780, *p* < 0.05), and wellbeing (*r* = 0.707, *p* < 0.05). There were also significant moderate positive correlations between the subscales and the between the subscales and subtotals, as illustrated in Figure 1. Each of the individual subtotals and subscales correlated positively and significantly (*p* < 0.05), with the exception of material wellbeing and emotional wellbeing (*r* = 0.131, *p* < 0.05).

### Quality of Life Outcomes

The age of the participants at the time of completing the POS was correlated with the total outcomes to investigate whether a relationship existed. There were no significant associations between participants' age and the total self- or proxy- outcome on the POS. There were also no significant differences noted when participants outcomes were compared on the total self-report POS (*p* = 0.575) or observer-report (*p* = 0.445) when stratified by gender. No significant difference was noted when participant's outcomes were compared when stratified by those who had a planner (*n* = 52), compared to those who did not (*n* = 33) for the self-report measure. However, observer-reports indicate that QoL was higher in those with a planner (*p* = 0.006).

Level of disability was considered categorically using MANOVA with Bonferroni *post-hoc* corrections made for multiple comparisons. Outcomes were stratified based on whether participants were within the mild, moderate, or severe/profound range historically i.e., not assessed or confirmed as part of this study. Participants with an intellectual disability in the mild range were reported to have a significantly higher total self-report than people with a severe/profound disability (*p* = 0.001) and not those with a moderate disability (*p* = 0.501). Individuals with a moderate disability were also found to have a significantly higher self-reported total score (*p* = 0.021). A similar pattern was reported for the total observer-report score (mild and severe/profound: *p* < 0.001; moderate and severe/profound: *p* = 0.014). When stratified based on whether a person was in gainful employment or not, there was a significant difference on both the self- and observer-report (*p* < 0.000001, respectively).

Participants were stratified based on the type of service they availed of e.g., residential, local, or outreach services; considering these groupings categorically using MANOVA with Bonferroni *post-hoc* corrections made for multiple comparisons, there were significant differences noted. Participants availing of local services had the lowest QoL for both the total self- and observer-report outcomes (117.32 ± 12.10 and 116.37 ± 11.17, respectively). This was significantly lower than those availing of outreach services (127.06 ± 7.98 and 126.68 ± 8.72: *p* = 0.007; *p* = 0.006), and residential services (123.09 ± 9.20 and 123.40 ± 11.19: *p* = 0.027; *p* = 0.025). There were no significant differences between those availing of residential compared to outreach services. In terms of living arrangements, each person availing of residential services resided at the host institution (*n* = 32; 100%); most participants availing of local services were living in the family home (*n* = 36; 97%), with 1 person living independently; and there was a near-even split between those living in the family home (*n* = 9; 56%) and those living independently (44%; *n* = 7) who attended outreach services. There were no significant differences in self- or observer-reported total outcomes on the POS when stratified by living arrangement. A breakdown of the mean and standard deviation for the total sample on the POS can be seen in Table 1, including the above stratifications.

**Table 1:** Outcome on the POS for the total group (N=85), with outcomes reported based on demographic and service-specific information.

**Category**	**Variable**	**N**	**Total self-report**	**Self-report: independence**	**Self-report: social participation**	**Self-report: wellbeing**	**Total observer-report**	**Observer report: independence**	**Observer report: social participation**	**Observer report: wellbeing**
**Total group**		N=85	121.32 ± 10.95	30.79 ± 3.81	41.35 ± 5.89	49.19 ± 3.48	120.96 ± 11.45	30.81 ± 3.93	41.13 ± 5.73	48.92 ± 3.71
**Gender**	Male	*n =* 41	120.63 ± 10.88	30.68 ± 3.38	41.12 ± 6.10	48.88 ± 3.48	119.97 ± 10.30	30.54 ± 3.89	41.02 ± 5.48	48.71 ± 3.51
	Female	*n =* 44	121.97 ± 11.09	30.89 ± 4.21	41.57 ± 5.66	49.48 ± 3.48	121.88 ± 12.47	31.07 ± 3.99	41.23 ± 6.00	49.11 ± 3.92
**Planner**	No	*n =* 33	118.00 ± 11.95	29.27 ± 4.20	40.76 ± 6.01	48.00 ± 3.69	116.72 ± 11.32	29.24 ± 4.02	39.55 ± 5.36	47.70 ± 3.57
	Yes	*n =* 52	123.44 ± 9.80	31.75 ± 3.23	41.73 ± 5.77	49.94 ± 3.14	123.65 ± 10.79	31.81 ± 3.56	42.13 ± 5.77	49.69 ± 3.62
**Disability**	Mild	*n =* 27	125.96 ± 6.96	32.76 ± 1.96	43.04 ± 4.31	50.16 ± 2.98	126.32 ± 7.97	33.48 ± 2.20	43.04 ± 4.88	49.96 ± 3.55
	Moderate	*n =* 48	121.64 ± 11.36	30.65 ± 4.05	41.13 ± 6.10	49.33 ± 3.55	121.02 ± 11.97	30.48 ± 3.91	40.92 ± 6.26	48.87 ± 3.74
	Severe/Profound	*n =* 10	111.20 ± 8.29	26.80 ± 3.04	39.70 ± 6.60	47.30 ± 2.66	109.70 ± 7.04	26.70 ± 3.52	37.90 ± 3.57	47.80 ± 2.82
**Service**	Residential Service	*n =* 32	123.09 ± 9.20	31.28 ± 3.37	41.06 ± 5.73	50.06 ± 3.29	123.40 ± 11.19	30.94 ± 3.83	41.59 ± 6.12	49.91 ± 3.65
	Local Service	*n =* 37	117.32 ± 12.10	29.41 ± 4.22	40.59 ± 6.10	47.95 ± 3.51	116.37 ± 11.17	29.59 ± 4.07	39.62 ± 5.28	47.84 ± 3.42
	Outreach Services	*n =* 16	127.06 ± 7.98	33.00 ± 2.16	43.69 ± 5.16	50.31 ± 3.00	126.68 ± 8.72	33.37 ± 2.36	43.69 ± 5.12	49.44 ± 4.04
**Living arrangements**	Community House	*n =* 32	123.09 ± 9.02	31.28 ± 3.37	41.06 ± 5.73	50.06 ± 3.29	123.40 ± 11.19	30.94 ± 3.83	41.59 ± 6.12	49.91 ± 3.65
	Family Home	*n =* 45	119.73 ± 12.49	30.16 ± 4.22	41.62 ± 6.36	48.47 ± 3.54	118.68 ± 11.80	30.36 ± 4.16	40.71 ± 5.79	48.27 ± 3.49
	Lives Independently	*n =* 10	123.25 ± 6.86	32.38 ± 2.32	41.00 ± 2.97	49.75 ± 3.32	124.00 ± 8.48	32.88 ±2.29	41.63 ±3.77	48.63 ±4.71

*The POS Total is scored out of a potential maximum score of 144. The Independence subscale has a maximum potential score of 36; The Social Participation subscale has a maximum potential score of 54, as does the Wellbeing subscale. The Self-report and Observer-report have the same scoring structure*.

## Discussion

The purpose of this study was to investigate outcomes on the POS from both a QoL and psychometric perspective in a community-sample of individuals with an intellectual disability in Ireland. Evidence would suggest that though proxy-report and self-report scores on measures of QoL demonstrate correlation, they do not provide identical information, and comparability may be reduced by external factors such as disability severity or instructions given to proxy respondents (18, 21, 22).

In this study, the correlation was both positive and strong between the total self-report and the total observed scores on the POS (*r* = *0.8*00; *p* < 0.01). Strong positive correlations were also observed between the self and observer reports on the subdomains (independence: *r* = *0*.913; *p* < 0.01, social participation: *r* = *0*.711, *p* < 0.01, and wellbeing *r* = *0*.693; *p* < 0.01). While the outcomes relate significantly to each other, a key consideration is that information from each respondent should be considered individually and as a complementary yet distinct data. Consequently, as a clinical consideration, the POS self-report interview should always be done where possible, and both outcomes should be considered independently from each other, rather than as a summed total score. Furthermore, our psychometric findings, based on the Cronbach's alpha of the individual eight QoL domains measured, would suggest that the three subdomains (independence, social participation, and wellbeing) should be considered as more reliable psychometric scales rather than the individual components themselves for both the self-report and observer-report. This may be particularly useful to consider if measuring change over time or the impact of an intervention. Each of the well-being components has a low Cronbach's alpha and low intra-factor correlations. This would suggest that well-being interventions should be considered holistically, and that supports need to be multifaceted to maximize the potential positive impact on well-being i.e., incorporating multi-element intervention e.g., emotional and physical well-being together (23).

Our findings are congruent with those from previous studies, which suggest that observer reports may score people with more severe intellectual disability lower on certain QoL domains than the person would score themselves (20); this may also be a reflection of family input for people who have more severe-to-profound intellectual disability. This is demonstrated in Table 1. This further highlights the importance of both self-report measures of QoL and the integration of proxy reports.

Simões and Santos (16) compared QoL for people with and without an intellectual disability in Portugal and found that living circumstances had a strong influence on QoL in terms of the rights domain, which is in line with our reported study. Also in line with our findings were those by a group in the Netherlands who reported that people who live more independently and are employed have a higher QoL (14). Specifically in our cohort, people who lived independently had the highest self-reported total score on the POS, as well as the highest total observer-report score. Additionally, adults availing of local services reported the lowest QoL scores, and 97% of these adults were living in the family home. These findings have clinical implications and suggest that living circumstances may have a strong impact on QoL for people with an intellectual disability. The correlation between living more independently and having a higher QoL may be due to enhanced independence and more opportunities to make personal decisions. This could be prospectively measured in the future. Furthermore, a family-based approach to improving QoL (24) could be a consideration for local (day) services, when considering QoL interventions for people with an intellectual disability who avail of their services (25–27).

A strength of this study is the consecutive recruitment, within a calendar year, of a large, well categorized, community-based sample, inclusive of many areas i.e., participants who avail of residential, local, and outreach services. This study is not without limitations. Firstly, the cross-sectional nature of the study does not allow for inferences to be made as to the degree of fluctuation in outcomes over time. Without clinically discreet categories, there is limited information at present as to what represents a “good” QoL or an outcome score or indeed what would represent meaningful or statistical change, both positively or negatively over-time or after specific intervention. A further limitation of the study and scale is the universal administration without modification for severity of disability, and so a person's outcome may be lower due to functional limitations, over and above a reduced QoL *per se*.

There are several prospective avenues for future research with this measure. The POS would benefit from the development and validation of clinically useful outcome ranges, which a baseline and follow-up intervention could be benchmarked against. Currently, in the interim, one could consider a 1, 1.5, and/or 2 standard deviations from the mean scores outlined in Table 1 as a mild (small), moderate (medium), or severe (large) deviation from this community-based normative sample. Further longitudinal measurements would be of benefit to further elucidate the test–retest reliability of the measure. Research could also investigate the clinical (mood), cognitive, and/or functional (activities of daily living) correlates of the POS, not only to better understand the relational properties of QoL to these outcomes, but to also consider potential avenues to improve, support, or maintain QoL (28). Based on the current study, the POS is shown to be a valid tool for measuring QoL for people with an intellectual disability, within the Irish healthcare system, when the total and subdomains are considered.

To conclude, this research investigated QoL outcomes on the POS and highlights that the subscales and total score are reliable indices. More research is needed to consider the clinical utility of the measure. This research shows that there are distinct and specific factors that are related to QoL for people with an intellectual disability in a community-based service, and future research could aim to investigate these factors longitudinally and specifically to determine how QoL relates to functional outcomes.

## Data Availability Statement

The raw data supporting the conclusions of this article will be made available by the authors, without undue reservation.

## Ethics Statement

The studies involving human participants were reviewed and approved by UCD HREC. Written informed consent for participation was not required for this study in accordance with the national legislation and the institutional requirements.

## Author Contributions

Each of the team were involved in the conceptual development of the manuscript. TB wrote the manuscript. MK, MV, WM, SB, and RB contributed to the data collection and collation. TB and OS completed the data analyses. RM, JL, and ML provided assessment measures. CD and OS contributed to the manuscript. All authors reviewed the manuscript.

## Funding

This work has been, in part, funded by the Irish Research Council under the New Foundations 2020.

## Conflict of Interest

The authors declare that the research was conducted in the absence of any commercial or financial relationships that could be construed as a potential conflict of interest.

## Publisher's Note

All claims expressed in this article are solely those of the authors and do not necessarily represent those of their affiliated organizations, or those of the publisher, the editors and the reviewers. Any product that may be evaluated in this article, or claim that may be made by its manufacturer, is not guaranteed or endorsed by the publisher.
